# BeiDou Short-Message Satellite Resource Allocation Algorithm Based on Deep Reinforcement Learning

**DOI:** 10.3390/e23080932

**Published:** 2021-07-22

**Authors:** Kaiwen Xia, Jing Feng, Chao Yan, Chaofan Duan

**Affiliations:** 1 Institute of Meteorology and Oceanography, National University of Defense Technology, Changsha 410005, China; xiakaiwenwen@nudt.edu.cn (K.X.); yanchao8302@163.com (C.Y.); duanchaofan18@nudt.edn.cn (C.D.); 2 Basic Department, Nanjing Tech University Pujiang Institute, Nanjing 211112, China

**Keywords:** BeiDou short-message, deep reinforcement learning, resource allocation, multi-objective optimization

## Abstract

The comprehensively completed BDS-3 short-message communication system, known as the short-message satellite communication system (SMSCS), will be widely used in traditional blind communication areas in the future. However, short-message processing resources for short-message satellites are relatively scarce. To improve the resource utilization of satellite systems and ensure the service quality of the short-message terminal is adequate, it is necessary to allocate and schedule short-message satellite processing resources in a multi-satellite coverage area. In order to solve the above problems, a short-message satellite resource allocation algorithm based on deep reinforcement learning (DRL-SRA) is proposed. First of all, using the characteristics of the SMSCS, a multi-objective joint optimization satellite resource allocation model is established to reduce short-message terminal path transmission loss, and achieve satellite load balancing and an adequate quality of service. Then, the number of input data dimensions is reduced using the region division strategy and a feature extraction network. The continuous spatial state is parameterized with a deep reinforcement learning algorithm based on the deep deterministic policy gradient (DDPG) framework. The simulation results show that the proposed algorithm can reduce the transmission loss of the short-message terminal path, improve the quality of service, and increase the resource utilization efficiency of the short-message satellite system while ensuring an appropriate satellite load balance.

## 1. Introduction

The global BeiDou Navigation System (BDS-3) is the fourth fully-fledged satellite navigation system to be developed after GPS, GLONASS, and Galileo. The BDS-3 has timing, positioning, and short-messaging services, and its unique short-messaging is widely used in meteorological and marine service, such as meteorological observation data collection [1,2], early warning information dissemination [3,4,5], and high-precision ocean measurements [6,7]. With the comprehensive completion of the BDS-3, the performance and service range of BDS-3 short-message communication (BDS3-SMC) have further improved, which has great practical significance for the more effective development of meteorological and marine service [8].

BDS3-SMC can provide regional short-message communication (RSMC) and global short-message communication (GSMC) [9]. The RSMC is served by three GEO satellites with large communication and service capacities, a low response delay (≤1 s), and high service frequency. GSMC is served by 14 MEO satellites, and its communication capacity and service capacity are significantly lower than those of RSMC. The GSMC processing resources for the satellite are scarce. For GSMC, on one hand, it is necessary to improve the resource utilization of the short-message satellite to ensure adequate system throughput. On the other hand, it is necessary to respond to the service requests of each terminal, provide the required services for the terminal, avoid uplink congestion, and shorten the request delay by up to hundreds of milliseconds. However, to determine how to reasonably allocate the global short-message processing resources of the BDS3-SMC, improve the resource utilization rate, and ensure the service quality of the short-message terminal, further study is required.

The existing satellite resource allocation (SRA) algorithms can be divided into traditional algorithms and artificial intelligence algorithms. Research involving the use of traditional optimization algorithms in satellite resource allocation is quite advanced. Developed algorithms include the genetic algorithm (GA) [10], simulated annealing algorithm (SA) [11], non-dominated sorting genetic algorithm (NSGA) [12], random geometry [13], and game theory [14,15]. Artiga et al. [16] established the satellite system power allocation optimization problem and used Lagrangian duality theory to optimize the total system capacity. Similarly, Choi et al. [17] applied Lagrangian theory to Karush–Kuhn–Tucker (KKT) conditions. Kan et al. [18] achieved multi-objective joint optimization of the energy efficiency (EE) and spectral efficiency (SE) of multi-beam satellites. At the same time, it was proven that the resource allocation problem in a multi-objective constraint scenario is an NP-hard problem. Therefore, heuristic algorithms such as the GA, SA, and NSGA can be widely used in satellite resource allocation scenarios. Aravanis et al. [19] proposed a multi-objective optimization strategy to minimize the power consumption of user terminals and satellites by using a meta-heuristic algorithm to reach a Pareto optimal solution. However, the calculation delay of the algorithm is long, and it is difficult to meet the requirements of real-time processing on the satellite using this method.

Based on the above problems, Efrem et al. [20] designed a continuous convex approximation algorithm to solve the multi-objective optimization problem of power distribution for energy-sensing of multi-beam satellites. This algorithm has a fast convergence speed and can be used for the dynamic allocation of satellite resources. By combining the particle swarm optimization algorithm and the Lyapunov optimization framework, Jiao et al. [21] solved the joint network stability and resource allocation optimization problems of high-throughput satellites (HTSs). Lin et al. [22] achieved joint optimization of wireless information resource allocation and power transmission of multi-beam solar satellites through particle swarm optimization (SPO), the improved harmony search algorithm (IHSA), and the monkey algorithm (MA), and analyzed SPO, ISHA, and MA algorithms. The results showed that the IHSA algorithm can maximize power transmission without affecting information transmission. However, the above work [20,21,22] did not consider the transmission power consumption and the quality of service of the task initiator.

There has been little research on short-message satellite resource allocation. Yang et al. [23] proposed a task-oriented satellite network resource allocation algorithm (SAGA) based on the GA. Xia et al. [24] combined this with the BDS3-SMC to form a short-message transmission mechanism and solved the problem of short-message satellite resource allocation by improving the Hungarian algorithm. However, for scenarios with a large number of terminals, the applicability of the algorithm is poor.

With the development of artificial intelligence technology, deep reinforcement learning (DRL) has made a substantial breakthrough in many tasks that need to interpret high-dimensional raw input data and implement sequential decision-making control [25]. Researchers have proven the effectiveness of DRL in many fields. Preliminary applications of DRL include resource allocation in the Internet of Things [26], heterogeneous cellular networks [27], and 5GHetNet uplink/downlink [28]; dynamic beam-hopping of satellite broadband systems [29]; and edge computing in the Internet of Things [30]. DRL has frequently been used in research work to optimize satellite resources. In fact, the SRA problem can be modeled as an interaction between the satellite system and the user terminal service, where the best solution to the problem is equivalent to the maximal cumulative reward that the agent (satellite system or user terminal) can get from the environment. In terms of multi-agent environments, DRL has been considered a solution for cognitive radio networks [31]. Ferreira et al. [32] proposed a reinforcement learning algorithm based on a deep neural network to solve the multi-objective optimization problem of resource allocation in cognitive satellite communication. Hu et al. [29,33,34] used DRL to make dynamic decisions for hopping beams in multi-beam satellite systems and next-generation broadband satellite systems, which have a lower level of complexity than traditional algorithms. He also proposed a resource allocation framework for multi-beam satellite systems based on DRL. In contrast, Luis et al. [35] proposed a dynamic satellite power allocation method based on DRL to minimize system power consumption. Yu et al. [36] proposed an optimization method to balance energy, power consumption, and efficiency in heterogeneous computing systems through reinforcement learning, and carry out hardware simulation experiments based on FGPA. The results show that reinforcement learning can greatly reduce system energy consumption without affecting hardware performance. Zhang et al. [37] proposed a multi-objective optimization algorithm based on deep reinforcement learning (DRL-MOP), which achieves multi-objective joint optimization of the satellite spectrum efficiency and improvements in energy efficiency and the service satisfaction index. Compared with the traditional GA and SA algorithms, it has been verified that the DRL-MOP algorithm has the characteristics of fast convergence and low complexity. Qiu et al. [38] proposed a software-defined satellite-terrestrial network (STN), which can be used to coordinate a satellite cache and computing resources, and can be combined with the DQN algorithm to optimize the cache and computing resources jointly.

Combined with the previous work in the field of multi-objective optimization, we find that DRL has surprising results in the field of multi-objective optimization. However, there is no relevant literature on the resource allocation of the SMSCS in the current research. Considering the actual scenario, the global short message resources of the SMSCS are very scarce. Due to the uneven distribution of short message terminals in various world regions (similar to IoT communication terminals, mobile phone terminals, etc.) it is reasonable to allocate the short message satellite processing resources as a critical way to improve the use efficiency of satellite resources and meet the needs of terminal services. Because of the above situation, the main work of this paper includes: (1) establishing a resource allocation model for the global short message satellite system of the SMSCS; and (2) proposing a resource allocation strategy to meet the needs of short message satellites and short message terminals.

According to the parameters of the short-message satellite communication system (SMSCS) [39], we first established a resource allocation model for the SMSCS. Furthermore, a resource allocation strategy for the BDS-3 short-message satellite is proposed with the optimization goals of improving the utilization of satellite resources and ensuring the service demands of the terminal are met. The resource allocation problem is described as a Markov decision process (MDP) and is solved by DRL.

The main contributions of the study are as follows.

(1) Based on the characteristics of the BDS3-SMC, an ideal SMSCS model is proposed. We formally describe the path transmission loss of the short-message terminal, satellite load balance, and satellite service quality through the above model and then establish a multi-objective optimization mathematical model for short-message satellite resource allocation.
(2) Considering that the number of short message terminals in the application scenario can reach more than one million, the huge input data makes DRL-SRA challenging to perform in the training process. We improve the ideal model of short-message satellite resource allocation and propose a region division strategy and a resource allocation model based on this strategy to reduce the computational complexity. The state space, action space, and reward mechanism of satellite resource allocation are defined according to the improved model.
(3) We design a feature extraction network to extract features from the state space to reduce the dimensions of the input data. Combined with the DDPG framework, it solves resource allocation in continuous states. Finally, we propose a BeiDou short-message satellite resource allocation algorithm based on DRL (DRL-SRA).

The rest of the paper is presented as follows. In Section 2 we introduce the system model. In Section 3 we optimize the proposed model and propose the DRL-SRA to solve the multi-objective optimization problem of short-message satellite resource allocation. In Section 4 we evaluate the performance of the proposed algorithm and the corresponding strategy through a simulation and compare it with the traditional algorithm and other reference strategies. In Section 5 we provide conclusions and present ideas for future work.

## 2. System Model

We consider the following scenario. In a snapshot [40] of an SMSCS, there are n short-message satellites (SAT) that cover the ground area; these are recorded as a set $SAT=\left\{SAT_{1},SAT_{2},\cdots ,SAT_{n}\right\}$, where n is the total number of short-message satellites in the SAT. At the same time, there are m short-message terminals (ST) in the coverage area; these are recorded as a set $ST=\left\{ST_{1},ST_{2},\cdots ,ST_{m}\right\}$, where m is the total number of short-message terminals in the ST. The communication link of the system adopts the Gaussian white noise channel, and the task requests of the short-message terminal have a Poisson distribution.

**Figure 1:**
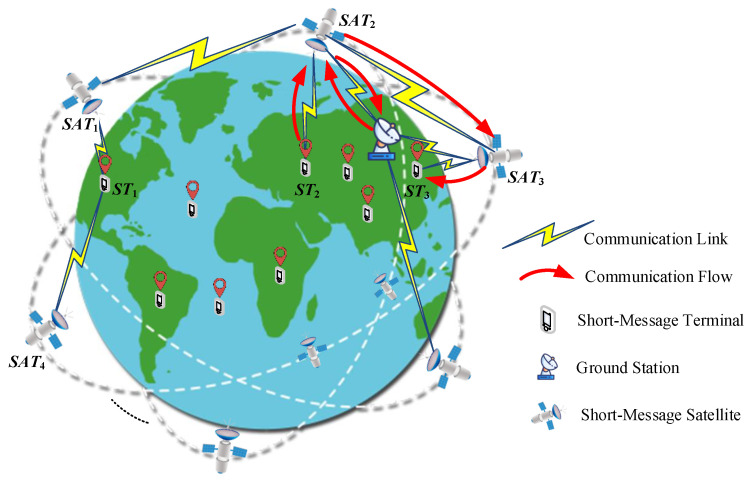
Short-message satellite communication system model.

Our system model is shown in Figure 1 and consists of the BDS-3 short-message satellite constellation, ground station, and short-message terminal.

The short-message satellite constellation adopted is the Walker 24/3/1 constellation, which is used to collect short-message transmission requests and return related information such as the working status to the ground station, and then wait for control commands from the ground station. The ground station can collect information on the working status, resource information, and transmission control instructions from the short-message satellite. The short-message terminal initiates task requests and receives short messages from other terminals.

The system workflow is divided into three stages. (1) The short-message terminal and the short-message satellite establish uplink and downlink communication links. (2) The short-message satellite establishes a communication link with the ground station, and the inbound information from the short-message terminal is sent to the ground station. (3) The short-message satellite sends the short message to the target terminal through itself or via the intersatellite link depending on the service instructions provided by the ground station. Short-message satellites can establish intersatellite links to achieve intersatellite information exchange. The red arrow in Figure 1 shows the end-to-end communication flow of a complete short-message terminal. First, $ST_{2}$ sends a short-message task request. Secondly, $SAT_{2}$ collects the request and informs the ground station, and the ground station sends the request to $SAT_{2}$ so that it can respond. Finally, $SAT_{2}$ transmits the short message from $ST_{2}$ to $ST_{3}$ through the intersatellite link established with $SAT_{3}$.

In the system model, the task requests from each short-message terminal can only be answered by a unique short-message satellite. Multiple short-message terminals can be distributed within the coverage area of each short-message satellite in the short-message satellite constellation. There is a one-to-many mapping relationship between a short-message satellite and the short-message terminal. As the short-message satellite has the characteristics of a large ground coverage area, the vast majority of short-message terminals are covered by multiple short-message satellites. Therefore, there is competition among short-message satellites to respond to short-message terminal tasks. For example, the task request of $ST_{1}$ shown in Figure 1 can be responded to by $SAT_{1}$ or $SAT_{4}$.

In different snapshots of the above model, due to the characteristics of short-message satellite coverage and the uneven distribution of short-message communication traffic in different regions, the resource utilization of the SMSCS and the energy efficiency of the short-message terminal are low. Consequently, our optimization objectives include the following:

(1) To reduce the transmission energy consumption of short-message terminals;
(2) To improve the resource utilization of short-message satellites;
(3) To produce adequate short-message terminal service quality.

To achieve these three optimization objectives, we formally describe the transmission energy consumption in the short-message terminal, the resource utilization of the satellite system, and the quality of service from the terminal.

**Definition** **1.** *Path transmission loss (L) is used to describe the transmission energy consumption of the short-message terminal, and it represents the transmission loss during the process of task transmission from the short-message terminal to the target satellite in dB.*

**Definition** **2.** *The load balancing index (LI) is used to describe the resource utilization rate of the satellite system. It indicates the degree of balance in task processing by short* *-message satellites, where the larger LI is, the higher the resource utilization rate of the satellite system will be.*

**Definition** **3.** *The service satisfaction index (SI) is used to describe the service quality of a short-message satellite transmitting to a short-message terminal. It indicates the efficiency at which a task is sent by the short-message satellite to the short-message terminal: the larger the SI, the higher the service quality.*

Additionally, the system model includes a communication model and a resource allocation model.

Communication model: The short-message terminal transmits the task request and content to the short-message satellite via the satellite link. The transmission delay is related to the size of the task data and the transmission rate. According to Shannon’s theorem, the communication model of $ST_{i}$ and $SAT_{j}$ can be defined as shown in Equation (1):

(1) $$r_{i,j}=B\log_{2}\left(1+\frac{P_{i}h_{i,j}}{\sigma^{2}+\sum_{m\in ST,m\neq i}P_{m}h_{m,j}}\right)$$

where i is $ST_{i}$, j is $SAT_{j}$, and m is $ST_{m}$, m∈ST but m≠i. $r_{i,j}$ represents the communication rate between $ST_{i}$ and $SAT_{j}$. B is the channel bandwidth. $\sigma^{2}$ is the communication noise power (Gaussian white noise). $P_{i}$ is the transmission power of $ST_{i}$, which can be approximated by the path transmission loss $L_{ij}$ between $ST_{i}$ and $SAT_{j}$, i.e., $P_{i}\approx L_{ij}$. $h_{i,j}$ is the channel gain between $ST_{i}$ and $SAT_{j}$. $P_{m}h_{m,j}$ is the interference to $ST_{i}$ caused by other short-message terminals.

**Figure 2:**
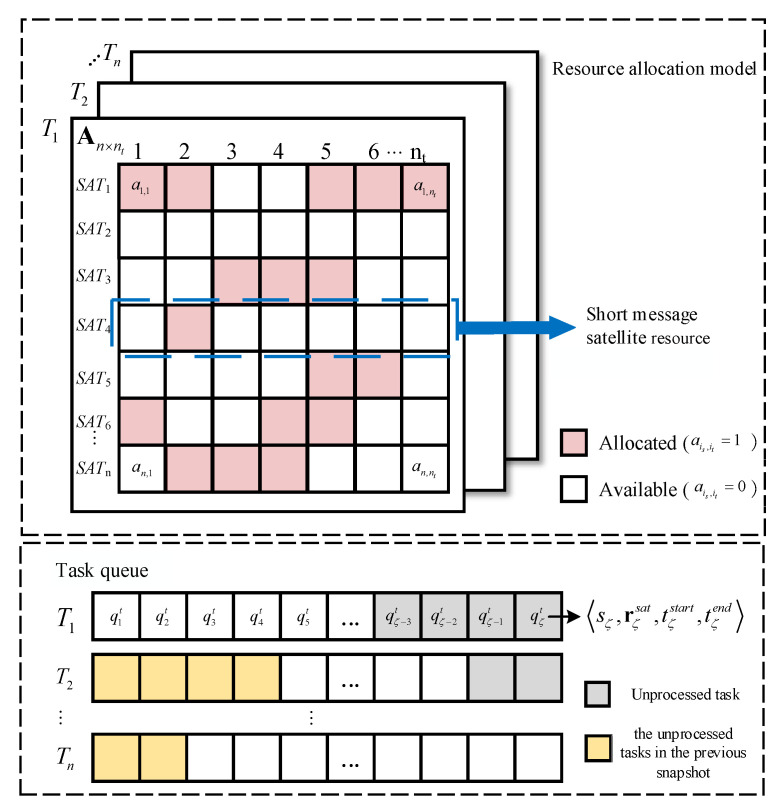
Short-message satellite communication system resource allocation model.

Resource allocation model: This is divided into the satellite resource allocation model and the task queue model, as shown in Figure 2.

For satellite resource allocation, a snapshot is divided into s time slices, where s is the number of time slices in a snapshot. The duration of a time slice is called a time unit, and the value of the time unit is determined by its actual application. Because the amount of data transmitted by the short-message task is limited, the maximum single transmission length of GSMC is 560 bits. In this article, a certain time unit is required for the short-message satellite to process the task of maximum length $C_{\max}$ (assuming that all satellites have the same task processing ability). Thus, in a snapshot, the short-message satellite resource can be formally described as resource matrix $A_{n\times s}$ with n×s. Each row in $A_{n\times s}$ represents the utilization of the short-message request processing resource $a_{i_{s}}$ that a satellite has over s time slices, $i_{s}\in \left\{1,2,\cdots ,n\right\}$. For element $a_{i_{s},i_{t}}$ in matrix $A_{n\times s}$, $i_{t}\in \left\{1,2,\cdots ,n_{t}\right\}$, $a_{i_{s},i_{t}}$ represents the resource utilization on time slice $i_{t}$ of $SAT_{i_{s}}$, where $a_{i_{s},i_{t}}\in \left\{0,1\right\}$. When $a_{i_{s},i_{t}}=0$, the resource is available. When $a_{i_{s},i_{t}}=1$, the resource has been allocated.

The task queue is shared by all short-message satellites following the first-in-first-out principle. The task queue is recorded as the set $Q^{T}=\left\{q_{1}^{t},q_{2}^{t},\cdots ,q_{\zeta }^{t}\right\}$, where ζ is the maximum capacity of the task queue $\zeta \in N^{+}$, and the element $q_{i}^{t}$ is defined as a quadruple:

(2) $$q_{i}^{t}≜\langle size_{i},r_{i}^{sat},t_{i}^{start},t_{i}^{end}\rangle$$

where $size_{i}$ is the short-message task size. $r_{i}^{sat}$ is the response matrix, which records the short-message satellites that can respond to the task. For example, when n=3, $r_{i}^{sat}=\left[0,1,1\right]$, which means that $SAT_{2}$ and $SAT_{3}$ can respond to $q_{i}^{t}$. $t_{i}^{start}$ records the time when the task enters the queue. $t_{i}^{end}$ records the time that the task is processed.

The task queue is updated after the end of each snapshot, i.e., s time slices. The unprocessed tasks in the previous snapshot are copied to the head of the queue, and new tasks continue to be received in the current snapshot, i.e., $Q_{t+1}^{T}\leftarrow Q_{t}^{T}-task^{r}+task^{new}$. $task^{r}$ indicates that the task has been responded to, and $task^{new}$ is the new task.

### 2.1. Path Transmission Loss Model

First of all, we discuss the path transmission loss L of the satellite-to-ground link between the short-message satellite and the short-message terminal. n×m satellite-to-Earth links can be established between the elements of set SAT and set ST, and the satellite-to-Earth link matrix $E_{m\times n}$ can be expressed as shown in Equation (3):

(3) $$E_{m\times n}=\left[\begin{array}{cccc} e_{11} & e_{12} & \cdots & e_{1n}\\ e_{21} & e_{22} & \cdots & e_{2n}\\ \vdots & \vdots & \ddots & \vdots \\ e_{m1} & e_{m1} & \cdots & e_{mn} \end{array}\right]$$

where $e_{ij}$ is a Boolean variable indicating the link relationship between $ST_{i}$ and $SAT_{j}$. When $e_{ij}=0$, $ST_{i}$ and $SAT_{j}$ do not establish a link relationship, but when $e_{ij}=1$, $ST_{i}$ and $SAT_{j}$ establish a link relationship.

Suppose the path transmission loss of the satellite-to-Earth link is L, there is:

(4) $$L=l_{f}+l_{rain}+l_{a}+l_{o}.$$

In Equation (4), L is the free-space path loss. $l_{rain}$ is the rain loss. $l_{a}$ is the atmospheric absorption loss, and its value is related to the antenna elevation angle φ at the transmitting terminal. $l_{o}$ denotes other losses. $l_{f}$ satisfies Equation (5):

(5) $$l_{f}=92.4+20\log f_{c}+20\log d.$$

**Figure 3:**
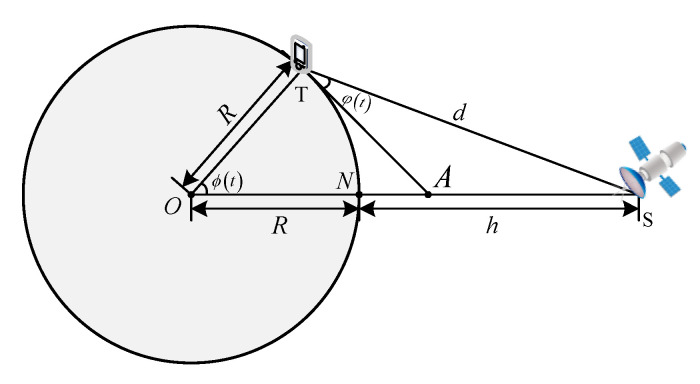
Geometric relationship between short-message terminal and satellite [43].

In Equation (5), d is the free-space transmission distance in km. f is the frequency in MHz. Generally, in the case of a fixed band, $l_{f}$ is only related to d. $l_{rain}$, $l_{a}$, and $l_{o}$ can be used to obtain the corresponding value of the band used in the current scene by consulting the related literature [41].

d can be obtained from geometric relations. Since the moving speed of the short-message terminal is very slow relative to the satellite speed, it can be assumed that the short-message terminal is motionless relative to the Earth [42]. In Figure 3, *O* is the geocenter, and *T* is the short-message terminal. *S* and *N* are, respectively, the position of the satellite and the sub-satellite point at time *t*. ϕ(t) is the geocentric angle between *T* and *N*. φ(t) is the elevation angle of the short-message terminal between *S* and *A*. *R* and *h* are the radius of the Earth and the orbital altitude, respectively.

For ϕ(t), there is:

(6) $$\phi \left(t\right)=\arccos\left(R\frac{\cos\varphi \left(t\right)}{h+R}\right)-\varphi \left(t\right).$$

Furthermore, the free-space transmission distance d is:

(7) $$d=\sqrt{{\left(h+R\right)}^{2}+R^{2}-2R\left(h+R\right)\cos\phi \left(t\right)}.$$

For n×m satellite-to-ground links, the path transmission loss matrix $L_{m\times n}$ between the elements of set SAT and set ST can be expressed as shown in Equation (8):

(8) $$L_{m\times n}=\left[\begin{array}{cccc} L_{11} & L_{12} & \cdots & L_{1n}\\ L_{21} & L_{22} & \cdots & L_{2n}\\ \vdots & \vdots & \ddots & \vdots \\ L_{m1} & L_{m1} & \cdots & L_{mn} \end{array}\right].$$

Then, the total path transmission loss $L_{total}$ that occurs when completing a message transmission in the current snapshot in the above set ST can be expressed as:

(9) $$L_{total}=\sum_{i=1}^{m}\sum_{j=1}^{n}l_{ij}e_{ji}.$$

### 2.2. Satellite Load Balancing Model

Satellite load balancing is an essential index for the rational utilization of satellite resources and the efficient processing of short-message tasks. We use the load balancing index LI to characterize the load balancing degree of the SMSCS.

Let $SAT_{j}$ respond to the number of short-message tasks ψ, recorded as the set $Task=\left\{task_{j1},task_{j2},\cdots ,task_{j\psi }\right\}$. A task $task_{j\psi }$ will continuously occupy $n_{j\psi }$ time slices. Since each satellite has, at most, $n_{t}$ time slices in a snapshot, the satellite resource utilization of $SAT_{j}$ in that snapshot is:

(10) $$SRR_{j}=\frac{\max\left(n_{t},\sum_{i=1}^{\psi }n_{ji}\right)}{n_{t}}$$

After the short-message satellite responds to the short-message task, the processing of each short-message does not affect the processing of others; they are processed in parallel. Therefore, for LI:

(11) $$LI=\frac{1}{m}{\sum_{j=1}^{m}\left(SRR_{j}-\frac{1}{m}\sum_{j=1}^{m}SRR_{j}\right)}^{2}.$$

Intuitively, the smaller LI is, the more balanced the load of the short-message satellite system is.

### 2.3. Terminal Satisfaction Model

In the previous section, the short-message tasks requested by m short-message terminals were recorded as the set $Task=\left\{task_{j1},task_{j2},\cdots ,task_{j\psi }\right\}$. We define the short-message task $task_{i}$ as a triple:

(12) $$task_{i}≜\langle c_{i},size_{i}^{t},\tau_{i}\rangle$$

where $c_{i}$ is the content of the short-message to be transmitted by $task_{i}$. $size_{i}^{t}$ is the size of the transmission task $task_{i}$ (in bytes). $\tau_{i}$ is the acceptable processing delay for $task_{i}$.

The satisfaction of the short-message terminal depends on the processing speed of the short-message satellite in response to the short-message terminal task request. The factors affecting the processing speed include the short-message transmission delay $t_{t,i}$, the processing delay $t_{p,i}$, and the task queuing delay $t_{q,i}$. Assuming that $SAT_{j}$ responds to the task request of $ST_{i}$, Equation (13) shows that the communication rate is $r_{i,j}$, the size of the transmission task $task_{i}$ is $s_{i}$, and the transmission delay is:

(13) $$t_{t,i}=\frac{size_{i}^{t}}{r_{i,j}}.$$

The processing delay is:

(14) $$t_{p,i}=\frac{size_{i}^{t}}{f_{j}}$$

where $f_{j}$ is the computing power of the short-message satellite, i.e., the number of bytes processed per unit of time.

The task queuing delay is:

(15) $$t_{q,i}=t_{i}^{end}-t_{i}^{start}-t_{p,i}.$$

Equations (13)–(15) show that the total execution delay for $task_{i}$ is:

(16) $$t_{i}^{\sum }=t_{t,i}+t_{p,i}+t_{q,i}.$$

If $ST_{i}$ does not request a short-message task, $t_{i}^{\sum }=0$.

Thus, the service satisfaction index for the short-message terminal set ST is:

(17) $$SI=\sum_{i=1}^{m}SI_{i}=\sum_{i=1}^{m}\mathrm{sgn}\left(t_{i}^{\sum }-\tau_{i}\right)$$

where $SI_{i}$ is the service satisfaction index of $ST_{i}$.

The main problem considered in this article is determining how to improve the resource utilization of the SMSCS, meet the quality-of-service requirements of the short-message terminal, and reduce the energy loss of the short-message terminal at the same time. In summary, the objective function of optimization under the snapshot t is:

(18) $$U\left(t\right)=\alpha_{1}\frac{L_{\min}}{L_{total}\left(t\right)}+\alpha_{2}\frac{LI\left(t\right)}{LI_{\max}}+\alpha_{3}\frac{SI\left(t\right)}{SI_{\max}}$$

where $\alpha_{1}$, $\alpha_{2}$, and $\alpha_{3}$ are weight values, and $\alpha_{1}+\alpha_{2}+\alpha_{3}=1$. $L_{\min}$ represents the minimum value of the overall path transmission loss of the short-message under the snapshot t. $LI_{\max}$ represents the maximum value of the load balance index. $SI_{\max}$ represents the maximum value of the terminal satisfaction index. The optimization objectives can be expressed as follows:

(19) $$\underset{L_{total}\left(t\right),LI\left(t\right),SI\left(t\right)}{\max}U\left(t\right)$$

(20) $$C1:\;\sum_{j=1}^{m}e_{ij}\leq 1,\;\forall i\in \left\{1,2,\cdots ,n\right\}$$

(21) $$C2:\;\sum_{i=1}^{\psi }s_{j,i}\leq \zeta C_{\max},\;\forall j\in \left\{1,2,\cdots ,m\right\}$$

(22) $$C3:P_{i}\geq l_{ij}e_{ji},\;\forall j\in \left\{1,2,\cdots ,m\right\},\;\forall i\in \left\{1,2,\cdots ,n\right\}$$

(23) $$C4:s_{i}\leq C_{\max},\;\forall i\in \left\{1,2,\cdots ,n\right\}$$

C1 is used to ensure that the short-message task of each short-message terminal in the communication system is answered by, at most, one short-message satellite. C2 is used to ensure that the number of short-message tasks answered by each short-message satellite does not exceed the maximum capacity it can handle. C3 is used to ensure that the energy consumed by the terminal transmission is not greater than the maximal amount of energy contained in the short-message terminal. C4 is used to ensure that the message size transmitted by the terminal does not exceed the maximal length specified by the system.

## 3. Algorithm Design

Based on the work of predecessors in multi-objective optimization, we propose DRL-SRA to solve the problem of short message satellite resource allocation. The area division strategy and the short message resource allocation algorithm based on the DDRG framework are used to solve two challenges: (1) Data preprocessing of SMSCS; and (2) DRL solves resource allocation in a continuous state.

### 3.1. Regional Division Strategy

The terminal capacity of the GSMS is about 1 million, and the terminal capacity of the RSMS is about 10 million. If the short-message satellites respond to the task requests of each short-message terminal, the calculation time complexity and space complexity will be high. Therefore, before designing the resource allocation algorithm, the system model needs to be optimized to reduce the overall overheads of the resource allocation algorithm.

**Figure 4:**
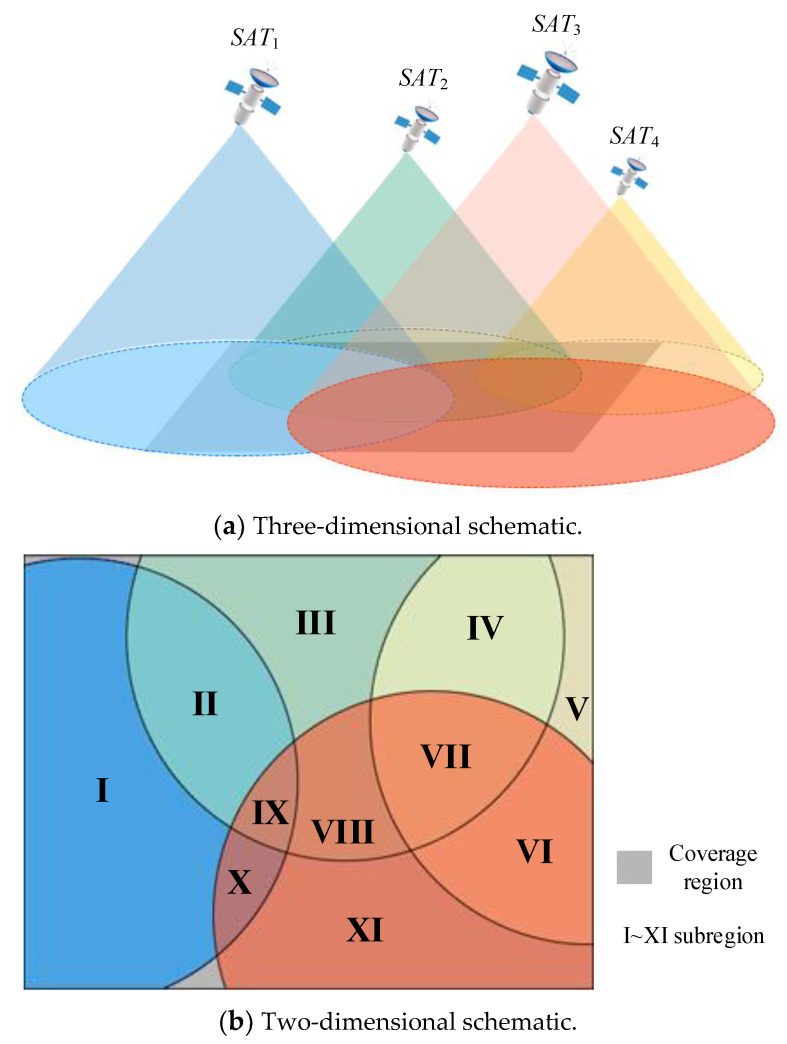
Schematic diagrams of short-message satellite coverage.

Because the BDS-3 MEO satellites use the Walker 24/3/1 constellation, an area is often covered by multiple short-message satellites. The coverage area is divided into υ subregions according to the type and number of covering satellites, and is recorded as the set $A^{r}=\left\{a_{1}^{r},a_{2}^{r},\cdots ,a_{\upsilon }^{r}\right\}$, where $a_{i}^{r}$ is the i-th subregion, and the maximum number of subregions that can be covered by a single short-message satellite is recorded as $\upsilon_{\max}$. The subregion is represented by a tuple, and for $a_{\upsilon }^{r}$, there is:

(24) $$a_{\upsilon }^{r}≜\langle C_{\upsilon }^{T},S_{\upsilon }\rangle$$

where $C_{\upsilon }^{T}$ is the number of short-message terminals included in subregion $a_{\upsilon }^{r}$. $S_{\upsilon }$ is the number of short-message satellites covering subregion $a_{\upsilon }^{r}$. As shown in Figure 4b, the coverage area can be divided into 11 subregions (because the Walker 24/3/1 constellation can achieve global coverage, the uncovered area in the schematic diagram is not discussed). The number of short-message satellites covered by subregions I, III, V, and XI is 1; therefore, the mission request of the short-message terminal in this region can only be responded to by the covered satellite. There are at least two short-message satellites in other subregions, so the optimal response scheme needs to be considered to satisfy Equation (19).

By introducing regional division, short-message terminals in a given subregion are regarded as a whole. The short-message satellite only needs to respond to the subregion, and does not need to respond to each short-message terminal separately. Thus, the satellite dynamic allocation problem of computation to the 10^6^–10^7^ power can be transformed into computation to the 10^2^–10^3^ power. However, the model proposed in the previous section needs to be improved further. The details are as follows.

The purpose of regional division is to approximate the number of short-message terminals in the subregion. Therefore, it is necessary to treat the short-message terminals in the subregion as a whole.

Firstly, considering the path transmission loss of a short-message terminal in a subregion, the following definition is given.

**Definition** **4.** *The regional distance is the average sum of distances of all the short-message terminals in the subregion from the target short-message satellite, i.e.,*

(25) $$A_{ij}^{d}=\sum_{\chi \in A}^{}d_{\chi j}$$

where $A_{ij}^{d}$ is the regional distance from $a_{i}^{r}$ to $SAT_{j}$, and $d_{\chi j}$ is the distance from $ST_{\chi }$ to $SAT_{j}$ in $a_{i}^{r}$.

Equation (5) shows that the path transmission loss $l_{ij}^{A}$ from the short-message terminal in $a_{i}^{r}$ to $SAT_{j}$ is:

(26) $$l_{ij}^{A}=\lambda \left(92.4+20\log f_{c}+20\log A_{ij}^{d}\right)$$

where λ is the number of $SAT_{j}$ responding to short-message task requests in $a_{i}^{r}$ and $\lambda \in N^{+}$.

The path transmission loss matrix ${\tilde{L}}_{\upsilon \times n}$ between the elements of set SAT and set $A^{r}$ can be expressed as shown in Equation (27):

(27) $${\tilde{L}}_{\upsilon \times n}=\left[\begin{array}{cccc} l_{11}^{A} & l_{12}^{A} & \cdots & l_{1n}^{A}\\ l_{21}^{A} & l_{22}^{A} & \cdots & l_{2n}^{A}\\ \vdots & \vdots & \ddots & \vdots \\ l_{\upsilon 1}^{A} & l_{\upsilon 2}^{A} & \cdots & l_{\upsilon n}^{A} \end{array}\right].$$

The total path transmission loss is:

(28) $${\tilde{L}}_{total}=\sum_{i=1}^{\upsilon }\sum_{j=1}^{n}\ell_{ij}l_{ij}^{A}$$

where $\ell_{ij}=1$ indicates that there is an intersatellite link between $a_{i}^{r}$ and $SAT_{j}$, and $\ell_{ij}=0$ indicates that there is no intersatellite link between $a_{i}^{r}$ and $SAT_{j}$.

Secondly, after completing regional division, each short-message satellite usually needs to serve multiple subregions; as shown in Figure 4, $SAT_{1}$ serves subregions I, II, IX, and X. Unlike the previous model, task requests in a subregion can be responded to by multiple satellites, and there are significant differences in the number and density of terminals in different subregions. Furthermore, each short-message satellite needs to allocate its own short-message processing resources to the subregions it covers. By using the resource allocation model proposed above, an improved satellite resource allocation model suitable for regional division can be obtained.

**Figure 5:**
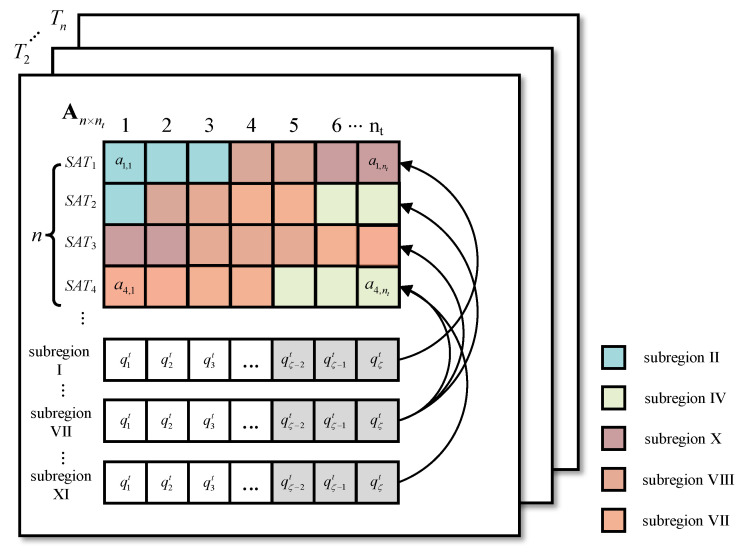
Improved satellite resource allocation model adapted for regional division.

As shown in Figure 5, a snapshot is divided into $n_{t}$ time slices, and each short-message satellite resource is allocated to a certain proportion of its covered subregion (the value of the subregion ratio is explained in detail in the next section). The color of the squares in Figure 5 corresponds to the colors of the subregions in Figure 4b, indicating how the resources are allocated in the current snapshot. Resources from different satellites obtained in the subregion are recorded as the set $R_{a^{r}}=\left\{R_{a^{r}}^{1},R_{a^{r}}^{2},\cdots ,R_{a^{r}}^{n}\right\}$, $a\in A^{r}$, where $R_{a^{r}}^{n}$ represents the resources allocated by $SAT_{n}$ to subregion $a^{r}$, and there is $\sum_{a^{r}\in A^{r}}R_{a^{r}}^{n}=1$.

Each subregion has a task queue, and its operation mode is similar to the resource allocation model mentioned in the second section. The difference lies in the task allocation in the task queue. In the improved model, based on the proportion of resources allocated by each satellite to the subregion, the tasks are divided among the covering satellites. For example, in subregion VII, the proportion of resources allocated by $SAT_{1}$ accounts for 45% of the resources obtained by subregion VII, so then 45% of the tasks in subregion VII are allocated to $SAT_{1}$. The unprocessed tasks in the current snapshot are copied to the next snapshot.

### 3.2. DRL-SAR Algorithm

When the short-message satellite receives a task request from short-message terminals in all subregions, it forwards the information, such as the short-message satellite status and the short-message terminal task request, to the ground station. The ground station determines the optimal strategy and returns the information to the short-message satellite system by using the satellite resource allocation algorithm to respond to the task request. Because the task request from the short-message terminal occurs randomly in the time dimension, the state transition probability of the system is difficult to calculate, and it is challenging to solve the problem by using the traditional value iteration method. The critical problem is determining the optimal strategy for allocating the short-message satellite response to short-message terminal tasks. As one of the basic methods of DRL, DQN is widely used in many optimization fields and can be used to effectively deal with tasks with a large state space and action space. However, because the output of DQN is discrete and the resource capacity of the short-message satellite and the energy of the short-message terminal are continuous variables, in order to meet the requirements for DQN input, the above continuous variables need to be quantized into discrete variables. This causes the action space to grow exponentially, which makes it challenging to guarantee the performance of DQN.

In order to solve the resource allocation problem of the short-message satellite system in continuous space, we propose a satellite short-message resource allocation algorithm based on deep reinforcement learning (DRL-SAR). The DRL-SAR algorithm takes Equation (18) as the optimization goal, models the short-message satellite as the agent, considers the response to the short-message terminal request as the action of the agent, and models the satellite-to-ground link as an interactive environment. The three elements of the DRL-SAR —status, action, and reward—can be described as follows.

(1) Status

Suppose the state space of the DRL-SAR is $S=\{s_{1},s_{2},\cdots ,s_{t}\}$, where $s_{t}$ is defined as the system state under snapshot t. For $s_{t}$ there is:

(29) $$s_{t}=\left\{L_{t},A_{n\times n_{t}}^{r},LI_{t}\right\}$$

where $L_{t}=\left\{L_{t,1},L_{t,2},\cdots ,L_{t,\upsilon }\right\}$. $L_{t,\upsilon }$ is the total path transmission loss of the terminal in subregion υ under $s_{t}$. $A_{n\times s}^{r}$ is the resource matrix under $s_{t}$, which involves the use information of satellite resources, such as resource occupancy and resource allocation. In $LI_{t}=\left\{LI_{t,1},LI_{t,2},\cdots ,LI_{t,\upsilon }\right\}$, $LI_{t,\upsilon }$ is the satisfaction degree of the terminal in area υ under $s_{t}$.

Since satellites and terminals are mobile, their levels of mobility are mapped as the change in distance between the subregion and the covered satellite and then further mapped to show the path transmission loss of the entire region. The mobility mentioned above only affects the state change of the DRL-SAR, but does not affect the overall framework design of the algorithm.

(2) Action

Suppose the action space is A, when all possible resource allocation decisions $b_{n}\left(t\right)$ of the satellite under $s_{t}$ are included:

(30) $$a_{t}=\{b_{1}\left(t\right),b_{2}\left(t\right),\cdots ,b_{n}\left(t\right)\}$$

where $b_{n}\left(t\right)$ is the resource allocation decision of $SAT_{n}$ under snapshot t. $b_{n}\left(t\right)=\left[p_{1,n},p_{2,n},\cdots ,p_{\upsilon ,n}\right]$, where $p_{i,n}$ is the proportion of resources allocated to subregion $a_{i}^{r}$ by the $SAT_{n}$ using its own resources, and there is $\sum_{i=1}^{\upsilon }p_{i,n}=1$. The allocation ratio has a continuous quantity, so it is necessary for the DRL-SAR to effectively deal with the continuous action space to solve the action dimension problem.

(3) Reward

Suppose that, under status $s_{t}$, the reward obtained by the system is $r(s_{t},a_{t})$.

Using Equation (18), the gain of the optimization objective can be expressed as:

(31) ΔU=U(t+1)−U(t).

For $r(s_{t},a_{t})$, there is:

(32) $$r(s_{t},a_{t})=\{\begin{array}{c} r_{h},\;\Delta U>0\;\\ r_{l},\;\Delta U\leq 0\; \end{array}.$$

When ΔU>0, the system revenue is increasing, $r(s_{t},a_{t})=r_{h}$. When ΔU≤0, the system revenue is unchanged or decreasing, $r(s_{t},a_{t})=r_{l}$, and $0\leq r_{l}<r_{h}\leq 1$.

**Figure 6:**
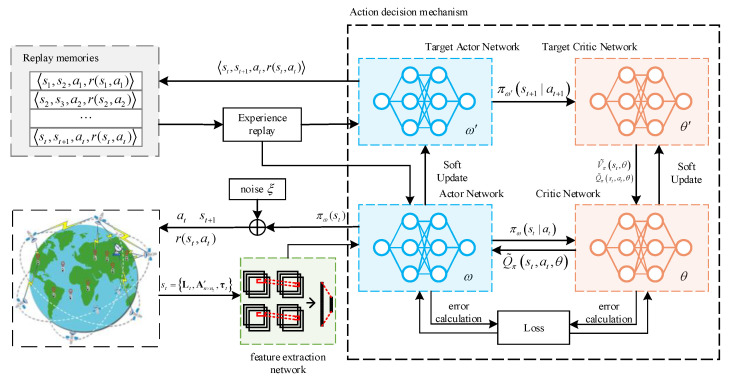
DRL-SAR algorithm framework.

For the short-message satellite resource allocation scenario, the proposed DRL-SAR framework is shown in Figure 6. The basic process is that the short-message satellite and the short-message terminal continuously interact to determine the current state of the environment and transmit environmental information to the ground station. Based on the state of the current system, the ground station sends the action instructions to the short-message satellite to be executed. After executing the instructions, the system environment moves from the current state to the next state and receives rewards through the environmental feedback. At the same time, the ground station stores the quadruple $\langle s_{t},s_{t+1},a_{t},r(s_{t},a_{t})\rangle$ as a sample in the memory pool, which is composed of the current environment state, the next state, executes actions and feeds back rewards. In the DRL-SAR training process, the training speed can be accelerated through experience replay.

The above algorithm framework is divided into two steps:

STEP 1: DRL-SAR input data reconstruction

The deep learning process carried out in the DRL-SAR involves the use of a feature extraction network to extract state features. The essence of the feature extraction network is the use of convolutional neural networks (CNNs). CNNs usually require input data to conform to the form of a graph tensor. For the state $s_{t}=\left\{L_{t},A_{n\times n_{t}}^{r},LI_{t}\right\}$, $L_{t}$, $A_{n\times n_{t}}^{r}$, and $LI_{t}$ are split into one-dimensional vectors, and $s_{t}$ is transformed into n+2 graphic tensors of α×α through operations such as zero padding and matrix transformation. The resource allocation of n short-message satellites and information about the path transmission loss of the terminal in the subregion and the service satisfaction of the short-message terminal are recorded. The graphic tensor outputs a one-dimensional vector with dimensions of $14\times \upsilon_{\max}$ through the feature extraction network. The one-dimensional vector records the characteristics of each short-message satellite for its covered subregion and inputs this information into the action decision mechanism.

STEP 2: DRL-SAR training and update

In state $s_{t}$ of the SMSCS, the ground station sends the instruction to execute action $a_{t}$. At this time, it receives a reward $r(s_{t},a_{t})$ and is transferred to state $s_{t+1}$. Assuming that the initial state of the SMSCS is $s_{0}$, strategy π is transferred from the initial state $s_{0}$ to the state $s_{t+1}$, as follows:

(33) $$\pi =\left\{\pi \left(s_{0}|a_{0}\right),\cdots ,\pi \left(s_{t}|a_{t}\right)\right\}.$$

In DRL-SAR, for $\pi \left(s_{t}|a_{t}\right)$, the action value function $Q^{\pi }\left(s_{t},a_{t}\right)$ is used to evaluate the benefits of action $a_{t}$ in the current state $s_{t}$ of the SMSCS. According to the Bellman equation, the action function is:

(34) $$Q_{\pi }^{}\left(s_{t},a_{t}\right)=r(s_{t},a_{t})+\gamma \sum_{s_{t+1}\in S}P\left(s_{t+1}|s_{t},a_{t}\right)V_{\pi }\left(s_{t+1}\right).$$

The state function is:

(35) $$V_{\pi }\left(s_{t}\right)=\sum_{a_{t}\in A}P\left(a_{t}|s_{t}\right)Q_{\pi }\left(s_{t},a_{t}\right)$$

where γ is the attenuation factor. $P\left(s_{t+1}|s_{t},a_{t}\right)$ is the probability that the SMSCS transfers to $s_{t+1}$ under state $s_{t}$ and action $a_{t}$. $P\left(a_{t}|s_{t}\right)$ is the probability of performing action $a_{t}$ under state $s_{t}$.

Equations (34) and (35) can be used to obtain the optimal action function $Q_{\pi }^{*}\left(s_{t},a_{t}\right)$ and the optimal state function $V^{*}\left(s_{t}\right)$:

(36) $$Q^{*}\left(s_{t},a_{t}\right)=r(s_{t},a_{t})+\gamma \sum_{s_{t+1}\in S}P\left(s_{t+1}|s_{t},a_{t}\right)\underset{a_{t+1}\in A}{\max}Q_{\pi }\left(s_{t+1},a_{t+1}\right)$$

(37) $$V*\left(s_{t}\right)=\underset{a_{t}\in A}{\max}Q^{*}\left(s_{t},a_{t}\right).$$

The optimal strategy is $\pi^{*}\left(s_{t}|a_{t}\right)$, the corresponding optimal action is $a_{t}^{*}$, and its expression is:

(38) $$a_{t}^{*}=\underset{a_{t}\in A}{\arg\max}Q_{\pi }^{}\left(s_{t},a_{t}\right).$$

Each snapshot in the SMSCS corresponds to the state action function $Q_{\pi }^{}\left(s_{t},a_{t}\right)$, the state function $V_{\pi }\left(s_{t}\right)$, and the optimal action $a_{i}^{*}$. The optimal strategy $\pi^{*}$ for transferring from the initial state $s_{0}$ to the state $s_{t+1}$ is:

(39) $$\pi^{*}=\left\{\pi^{*}\left(s_{0}|a_{0}\right),\cdots ,\pi^{*}\left(s_{t}|a_{t}\right)\right\}.$$

However, it is usually challenging to determine the state transition probability of the SMSCS, the state of the resource allocation problem is continuous, and the scale of the state set is large. We include the DDPG in the action decision mechanism of the DRL-SAR. Through the introduction of actor–critic, the continuous spatial state is parameterized.

First, by introducing ${\tilde{V}}_{\pi }$ and ${\tilde{Q}}_{\pi }$, the state function and the action function are approximated. They are:

(40) $${\tilde{V}}_{\pi }\left(s_{t},\theta \right)\approx V_{\pi }\left(s_{t}\right)$$

(41) $${\tilde{Q}}_{\pi }\left(s_{t},a_{t},\theta \right)\approx Q_{\pi }^{}\left(s_{t},a_{t}\right).$$

Similarly, to approximate the strategy function, we have:

(42) $$\pi_{\omega }\left(s_{t}|a_{t}\right)=P\left(a_{t}|s_{t},\omega \right)\approx \pi \left(s_{t}|a_{t}\right)$$

where θ and ω are the weight parameters in the network.

DDPG includes four networks, namely, the target critic network, the critic network, the target actor network, and the actor network. The basic idea is that the strategy gradient is approximated by the strategy function and the value function. In this process, the strategy function can evaluate and optimize the strategy based on the value function. The optimized strategy function can also make the value function reflect the value of the state more accurately, and the functions can influence each other to obtain the optimal solution [44]. The actor network has a policy function and is responsible for agent selection and environment interactions. The critic network has a value function and is used to evaluate the behavior of the actor. In the DRL-SAR, the main functions of the four networks are as follows.

(1) The target critic network is responsible for calculating targets ${\tilde{V}}_{\pi }\left(s_{t+1},\theta^{\prime }\right)$ and ${\tilde{Q}}_{\pi }\left(s_{t+1},a_{t+1},\theta^{\prime }\right)$ based on the state sampled in the experience replay pool. Parameter $\theta^{\prime }$ in the target critic network is regularly copied from θ in the critic network, i.e.,
  (43) $$\theta^{\prime }=\mu \theta +\left(1-\mu \right)\theta^{\prime }$$
  where μ is the updated coefficient, and 0<μ≪1.
(2) The critic network is responsible for iteratively updating parameter θ in the value function and calculating the current values of ${\tilde{V}}_{\pi }\left(s_{t},\theta \right)$ and ${\tilde{Q}}_{\pi }\left(s_{t},a_{t},\theta \right)$. The loss function of the critic network can be defined as:
  (44) $$J\left(\theta \right)=\frac{1}{N}\sum_{i=1}^{N}{\left[r(s_{t},a_{t})+\gamma {\tilde{Q}}_{\pi }\left(s_{t+1},a_{t+1},\theta^{\prime }\right)-{\tilde{Q}}_{\pi }\left(s_{t},a_{t},\theta \right)\right]}^{2}$$
  where N is the number of samples drawn from the experience playback pool and N>0.
(3) The target actor target network is responsible for selecting the optimal action $a_{t+1}$ based on the state $s_{t+1}$ sampled in the experience replay pool. The parameter $\omega^{\prime }$ in the target actor target network is periodically copied from ω in the actor network, i.e.,
  (45) $$\omega^{\prime }=\mu \omega +\left(1-\mu \right)\omega^{\prime }.$$
(4) The actor network is responsible for iteratively updating parameter ω in the strategy function. According to the state $s_{t}$ of the SMSCS in the current snapshot t, it selects the current action $a_{t}$, obtains the reward $r(s_{t},a_{t})$, and determines the initial state $s_{t+1}$ of snapshot t+1. The loss of the actor network can be simply understood as follows: the greater the value of the action obtained, the smaller the network loss. Therefore, the loss function of the actor network can be defined as:
  (46) $$J\left(\omega \right)=-\frac{1}{N}\sum_{i=1}^{N}{\tilde{Q}}_{\pi }\left(s_{t},a_{t},\theta \right).$$

The loss functions J(θ) and J(ω) use gradient direction propagation to update the neural network parameters. At the same time, they balance the exploration of new actions and the use of known actions, increase the randomness of the learning process, and improve the generalization ability of the DRL-SAR. We add random noise ξ to action $a_{t}$ obtained by the actor network in state $s_{t}$, which is given by:

(47) $$a_{t}=\pi_{\omega }(s_{t})+\xi$$

In summary, the DRL-SAR is shown as Algorithm 1.
**Algorithm 1** DRL-SAR**Input:**ω, θ, $S=\{s_{1},s_{2},\cdots ,s_{t}\}$ **Output: Optimization result** **Begin** 1:Initialize the actor network $\pi_{\omega }\left(s_{t}|a_{t}\right)$ and critic network ${\tilde{Q}}_{\pi }\left(s_{t},a_{t},\theta \right)$ with weights ω and θ2:Initialize the target actor network $\pi_{\omega^{\prime }}\left(s_{t}|a_{t}\right)$ and target critic network ${\tilde{Q}}_{\pi }\left(s_{t},a_{t},\theta^{\prime }\right)$ with weights $\omega^{\prime }$ and $\theta^{\prime }$ with initial weights $\omega^{\prime }=\omega$ and $\theta^{\prime }=\theta$3:Initialize the parameters and initial states of n short-message satellites and m short-message terminals4:Initialize the replay memories D, weight update interval, and random noise ξ5:Initialize the state space $S=\{s_{1},s_{2},\cdots ,s_{t}\}$ and $s_{t}=\left\{L_{t},A_{n\times n_{t}}^{r},\tau_{t}\right\}$, and get the graph tensor $s_{1},s_{2},\cdots ,s_{t}$6:**for** i **in** range($T_{max}$):7: Get $a_{t}=\pi_{\omega }(s_{t})+\xi$ in $S=\{s_{1},s_{2},\cdots ,s_{t}\}$.8: Get $s_{t+1}$ and $r(s_{t},a_{t})$ by $a_{t}$.9: Store transition $\langle s_{t},s_{t+1},a_{t},r(s_{t},a_{t})\rangle$ in replay memories D10:  **if** len(D)>z:11:  Randomly sample a batch of experiences $\tilde{D}$ from D12:  Calculate ${\tilde{Q}}_{\pi }\left(s_{t+1},a_{t+1},\theta^{\prime }\right)$13:  Update θ according to Equation (43)14:  Update ω according to Equation (44)15:  **end if**16:  **if** $T_{max}\mathrm{mod}\;n_{0}==1$:17:  $\theta^{\prime }=\mu \theta +\left(1-\mu \right)\theta^{\prime }$18:  $\omega^{\prime }=\mu \omega +\left(1-\mu \right)\omega^{\prime }$19:  **end if**20:  **end for**21:The model iteration ends and the optimization result is returned **End**

## 4. Simulation and Performance Analysis

This section describes the evaluation of the performance of the algorithm proposed in this article for different system parameters. To verify the effectiveness and convergence of the DRL-SAR, we used the throughput and load balance index of the SMSCS, the path transmission loss of the short-message terminal, and the terminal service satisfaction index as the algorithm evaluation criteria. At the same time, the DRL-SAR was compared with the DQN, GA, and TS-IHA [24]. An Intel(R) Xeon(R) W-2104 CPU @3.20 Hz, 16 GB RAM computer was used for the simulation experiments conducted in this work. The simulation platform was based on Python 3.6, and the neural network in the DRL-SAR was built through TensorFlow.

### 4.1. Simulation Parameter Setting

The SMSCS uses MEO satellites with an orbital altitude of 21,528 km and an orbital inclination of 55°. It is distributed in the Walker 24/3/1 constellation, which contains a total of 24 satellites, of which 14 provide GSMC. The scene was built with STK satellite simulation software.

**Figure 7:**
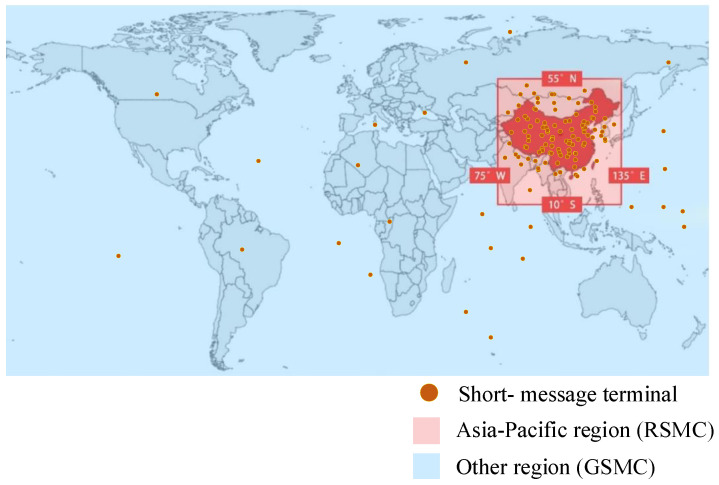
Schematic diagram of the short-message terminal distribution.

Considering future practical application scenarios, 1000 short-message terminals were randomly placed in the Asia-Pacific region at 55° N–10° S and 75° W–135° E (the red area in Figure 7). In the other region, 100,400 short-message terminals were randomly placed.

The short-message terminals in the two areas can randomly choose whether or not to send short-message requests in each snapshot. The size of short-messages transmitted by the short-message terminals obeys a normal distribution with an expectation of $\frac{C_{\max}}{2}$ and a variance of 1.

The network design consists of two parts. The feature extraction network includes two convolution layers (Conv) and three FC layers (FC), which were used to extract the features of the graph tensor. The specific parameters are shown in Table 1.

**Figure 8:**
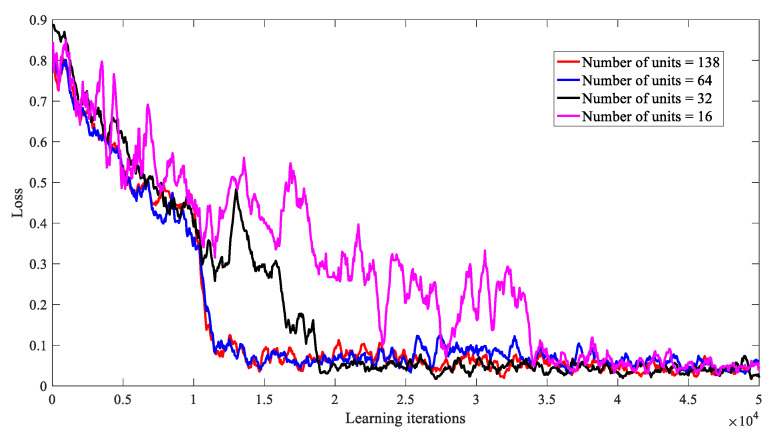
Convergence rate under different numbers of hidden layer units.

The four DDPG networks have the same network structure, in which there are three hidden layers. The loss function adopts the Relu function, and the number of neurons in each layer were set to 16, 32, 64, and 128, respectively. First of all, the deep neural networks with different structures were run to analyze the training efficiency, as shown in Figure 8. The abscissa represents the number of iterations, and the ordinate represents the average loss of the network after continuous learning. The average loss was obtained by exponential moving average (EMA) smoothing, and the smoothed curve better reveals the changing trends of the data. The results show that deep neural networks with different neurons converge after 35,000 iterations of training. However, the structure of 64 neurons in each layer can ensure the minimum network parameters are met to ensure the best convergence performance is achieved. Therefore, we set the number of neurons in the hidden layers to 64, which can also be used as the training structure for the next part of the network performance evaluation.

**Figure 9:**
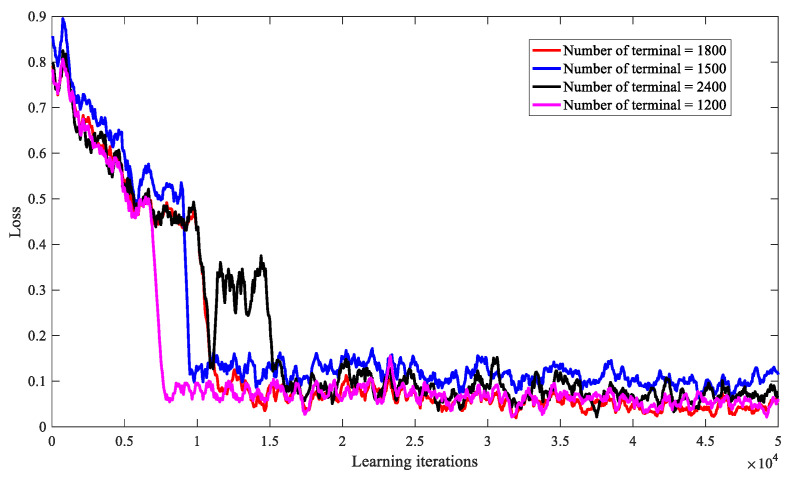
Convergence of DRL-SRA algorithm under different numbers of short message terminals.

At the same time, we analyze the scalability of DRL-SRA when the number of short message terminals is 1200, 1500, 1800, and 2400, and the impact on the performance of the DRL-SRA algorithm. As shown in Figure 9, it can be found that when the number of short message terminals takes different values, the algorithms can all converge. Moreover, the smaller the number of short message terminals, the faster the convergence speed of the algorithm, but the approximate convergence state can be obtained in the end, indicating that the algorithm has good scalability.

**Figure 10:**
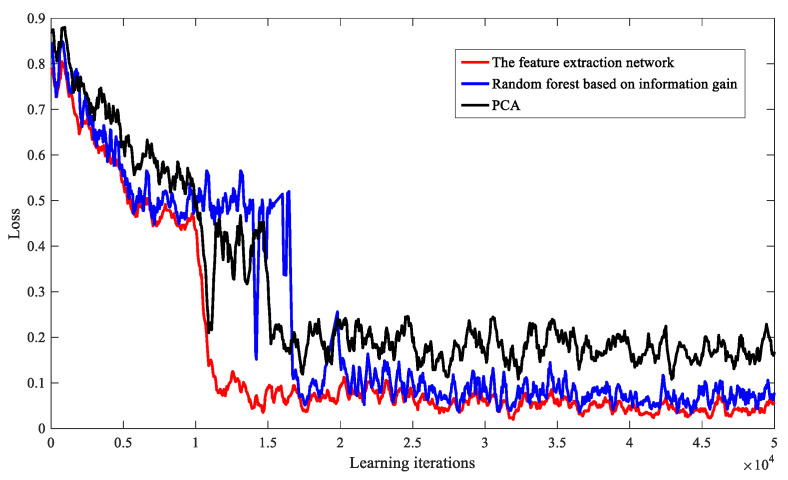
Convergence of DRL-SRA algorithm under different feature extraction algorithms.

Finally, when the number of terminals is 1500, we compare the performance of the feature extraction network with the PCA dimensionality reduction model and the random forest feature extraction model based on information gain. As shown in Figure 10, the comparison results show that the algorithm using the feature extraction network has a better convergence effect during training.

The specific simulation parameters are shown in Table 2.

### 4.2. Analysis of Simulation Results

#### 4.2.1. Algorithm Performance Comparison

We compared the DRL-SRA with the DQN, TI-SHA, and GA. Using different numbers of short-message terminals, the optimization effects of the total path transmission loss ($L_{total}$), load balancing index (LI), and service satisfaction index (SI) were analyzed. The comparison algorithm is described as follows:

(1) DQN: The Q network and target Q network with the same network structure are included, the Q network has three hidden layers, and the number of neurons in each layer is 64. The DQN strategy is shown in Equation (38).
(2) TS-IHA: The Hungarian algorithm is satisfied by adding a virtual satellite and terminal.
(3) GA: The population size is 50, the termination evolution number is 400, the crossover probability is 0.9, and the mutation probability is 0.01.

The above four algorithms have the same weights for the three objectives, i.e., $\alpha_{1}=\alpha_{2}=\alpha_{3}=\frac{1}{3}$.

In addition, after the training has been completed, the network parameters in DRL-SRA are not updated in subsequent experiments.

**Figure 11:**
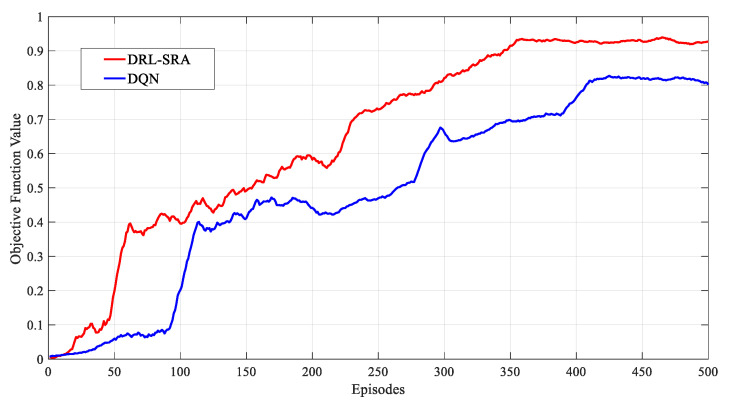
Convergence comparison of the DRL-SRA and DQN.

First of all, we evaluated the convergence of the proposed algorithm. Figure 11 shows the convergence effect of the DRL-SRA when the number of short-message terminals was 1500, the number of training steps was 500, and the number of iterations was 500. The abscissa shows the number of iterations, and the ordinate shows the objective function for Equation (18), which is compared with the DQN. In the process of comparison, the EMA was also used to smooth each original data curve for these objective function values. As shown in Figure 11, the objective function value curves of the two algorithms grew at different rates as the training process proceeded. In the first 50 iterations of DRL-SRA, the value of the objective function quickly reached 0.492. After 350 iterative periods, the value of the objective function increased to 0.957, obtaining a state of convergence. The value of the objective function of the DQN reached 0.823 in the first 400 iterations and did not continue to grow. At the end of the training period, the DRL-SRA algorithm had a higher objective function value than the DQN algorithm; that is, a better convergence performance.

**Figure 12:**
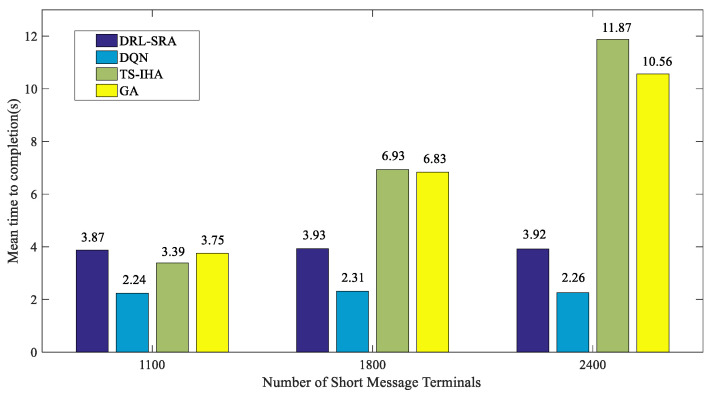
Comparison of the running times of different algorithms.

Furthermore, the running times of the four algorithms were compared. As shown in Figure 12, the average time taken by the four algorithms to complete a strategy selection process was calculated when there were 1100, 1800, and 2400 short-message terminals.

When there were 1100, 1800, and 2400 short-message terminals, the average completion time for the DRL-SRA algorithm was 3.87 s, 3.93 s, and 3.92 s, respectively. The average completion time for the DQN is slightly lower than that of the DRL-SRA because there are fewer network parameters in the DQN compared with the DRL-SRA. With an increase in the number of terminals in the TS-IHA and GA, the time required to run the algorithm obviously increased. However, the time consumed by the DRL-SRA and DQN remained unchanged, which shows that the average completion time of decision actions input into the DRL-SRA and DQN is determined by the parameters of the network (such as the number of network layers). In contrast, the network parameters are fixed after the DRL-SRA and DQN complete the training period.

Finally, to illustrate the performance of the DRL-SRA, we set the weight factor to $\left(\alpha_{1}=\frac{1}{3},\alpha_{2}=\frac{1}{3},\alpha_{3}=\frac{1}{3}\right)$ and analyzed the optimization of $L_{total}$, SI, and LI using the four algorithms.

**Figure 13:**
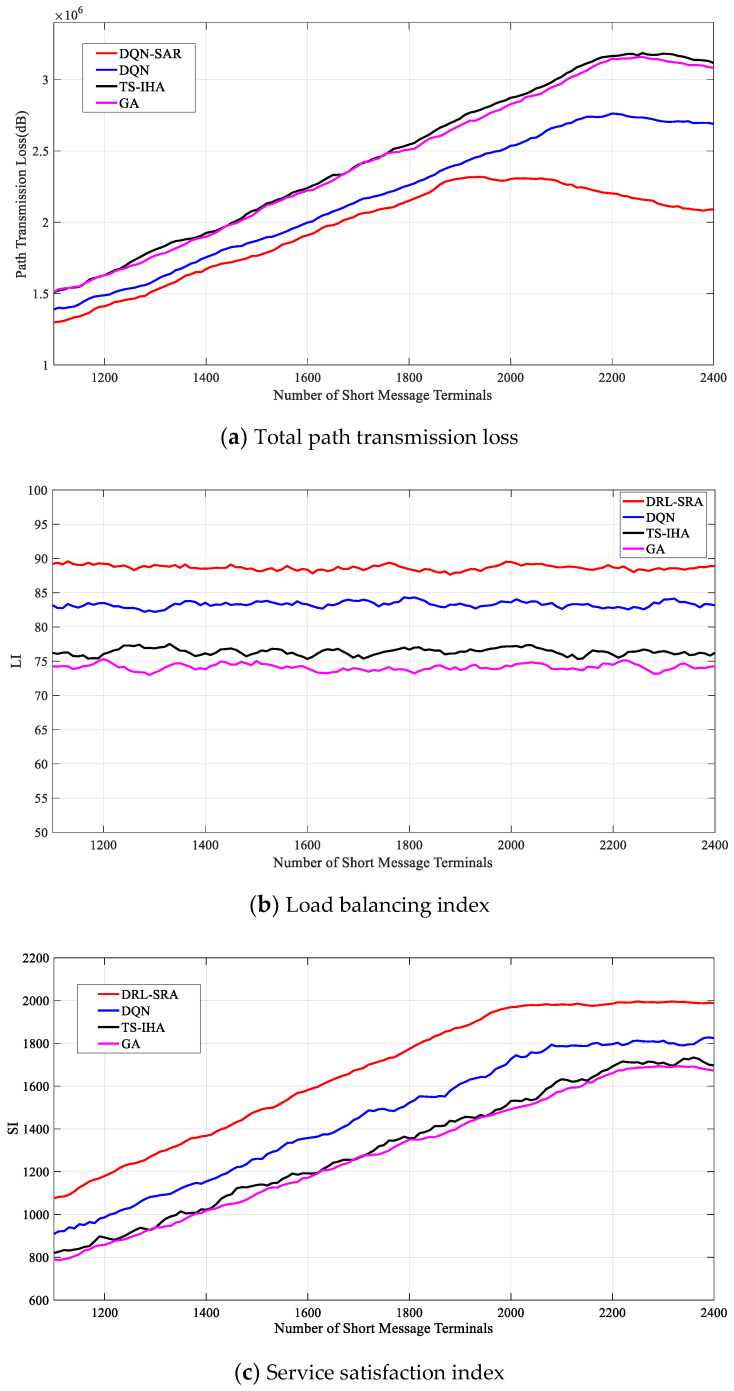
Performance analysis and comparison of different algorithms.

As shown in Figure 13, according to the deployment requirements of the short-message terminals described above, as the number of short-message terminals increased from 1100 to 2400, the $L_{total}$ obtained by different algorithms showed a trend of rising at first and then decreasing. SI basically remained stable, and LI showed a trend of growing at first and then becoming steady. This is in line with the expected effects. With an increase in the number of short-message terminals, the number of short-message tasks received by the short-message satellite also increased, and L also increased. However, the number of tasks increased to a certain extent because the choice of tasks that the satellite can respond to will also increase when both SI and LI remain stable, and $L_{total}$ will slowly decrease. At the same time, when the short-message satellite resources are fixed and when the number of short-message tasks reaches a certain threshold, the task processing capacity of the short-message satellite reaches saturation, leading to the inability to respond to more short-message tasks in time.

Figure 13a shows that the DRL-SRA performed the best and the TS-IHA performed the worst. The $L_{total}$ of the DRL-SRA was lower than that of the other three algorithms in the simulation process. Figure 13b shows that DRL-SRA performed the best and GA performed the worst. When the number of terminals was 1100 or 2250, the LI obtained by the DRL-SRA algorithm was about 89.14%, which is higher than the values obtained by the other algorithms: 83.58% by the DQN algorithm, 76.37% by the DQN, and 73.83% by the GA. Figure 13c shows that the DRL-SRA performed the best and the GA performed the worst. When the number of short-message terminals was 2050, the SI obtained by the DRL-SRA was about 2000, which is higher than the value of 1800 obtained by the DQN algorithm when the number of short-message terminals was 2100 and the value of 1700 obtained by the TS-IHA algorithm when the number of short-message terminals was 2250.

#### 4.2.2. The Influences of Different Weight Values on the Optimization Results

**Figure 14:**
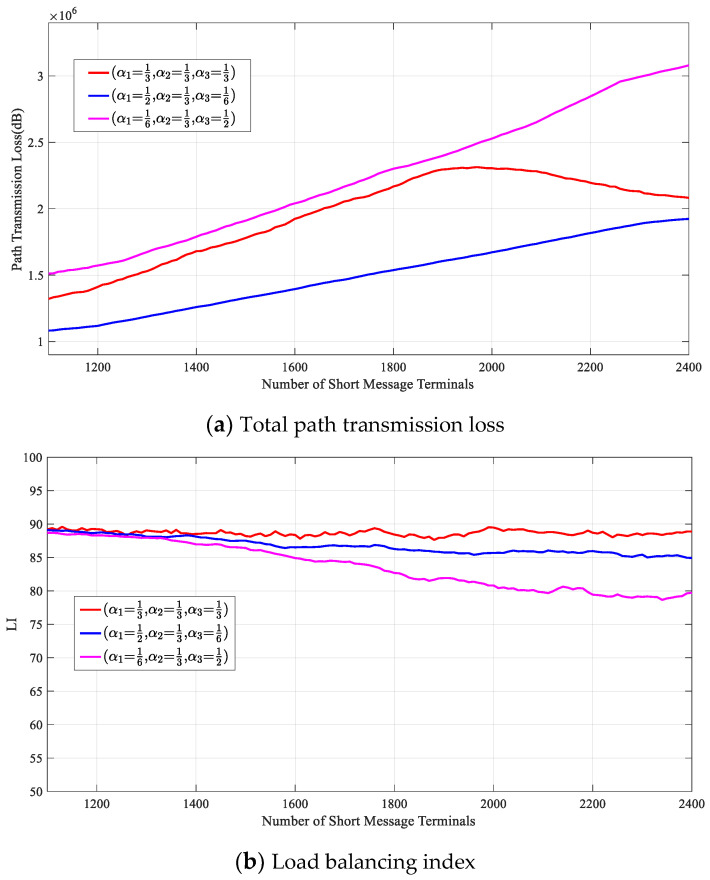

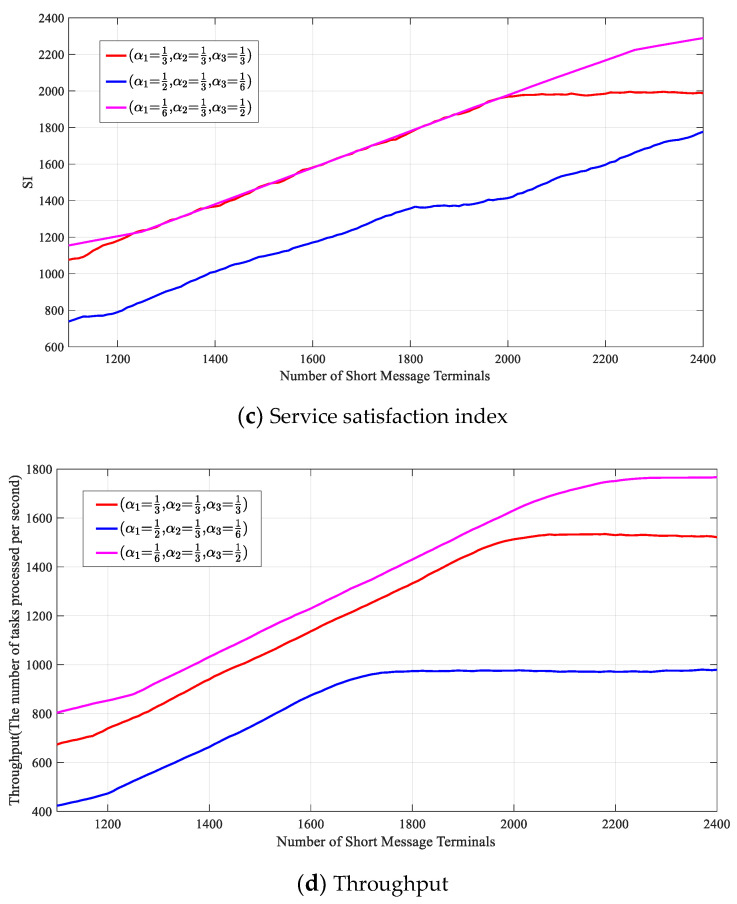
Performance analysis of the DRL-SRA with different weight values.

Figure 14 shows the optimization effect of the DRL-SRA on the throughput of the short-message satellite system and $L_{total}$, LI, and SI when the weight factors of $L_{total}$, LI, and SI were $\left(\alpha_{1}=\frac{1}{3},\alpha_{2}=\frac{1}{3},\alpha_{3}=\frac{1}{3}\right)$, $\left(\alpha_{1}=\frac{1}{6},\alpha_{2}=\frac{1}{3},\alpha_{3}=\frac{1}{2}\right)$, and $\left(\alpha_{1}=\frac{1}{2},\alpha_{2}=\frac{1}{3},\alpha_{3}=\frac{1}{6}\right)$, respectively.

As shown in Figure 14d, as the number of short-message terminals increased, the total throughput of each algorithm first increased and then stabilized. Because the user service arrival model and service requirements used in the simulation were the same, when the weight parameter was $\left(\alpha_{1}=\frac{1}{6},\alpha_{2}=\frac{1}{3},\alpha_{3}=\frac{1}{2}\right)$ and the number of terminals was 2250, the throughput of the system was 1790, which was the best because the weight of LI was relatively high. To improve the quality of service of the short-message terminal as much as possible, the DRL-SRA will have a greater requirement for the response time to the short-message request. This can also be proven by looking at the satellite load balance shown in Figure 14b. To improve the quality of service of the short-message terminal, the utilization of satellite resources needs to be maximized, so when the weight of LI is less than that of SI, LI is not significantly reduced relative to other weights.

When the weight parameter is $\left(\alpha_{1}=\frac{1}{2},\alpha_{2}=\frac{1}{3},\alpha_{3}=\frac{1}{6}\right)$, the system will reduce $L_{total}$ as much as possible, which will reduce the efficiency of short-message task processing, so the quality of service of the terminal in this set of weight parameters will be reduced relative to other weights. Additionally, the short-message satellite will respond to the task request as accurately as possible, which will make it challenging to achieve a relative balance in the use of satellite resources in the scenario of an uneven distribution of short-message terminals. LI is significantly lower than other weights.

## 5. Conclusions

In this paper we focused on the existing conditionally constrained short-message satellite resource allocation model. In order to reduce the path transmission loss of the SMSCS and maximize the satellite load balancing and terminal service quality, a multi-objective optimization mathematical model was proposed. Due to the large number of terminals and problems with the action dimension, we proposed a region division strategy and the DRL-SRA algorithm based on the feature extraction network and DDPG. This method can achieve dynamic multi-objective optimization and resource allocation with a low level of complexity. By simulating a real application scenario, DRL-SRA was shown to be more effective than traditional algorithms for optimizing the path transmission loss of the short-message terminal and maintaining the load balance and quality of service of the short-message satellite. When there are different numbers of terminals, the DRL-SRA can send better results. The simulation results also show that, compared with other algorithms, the DRL-SRA has a better effect on improving the throughput of short-message task requests.

In future work, we will combine the short-message terminal equipment and the low-power computing chip that supports the deep learning algorithm to consider the hardware implementation flow of the DRL-SRA. We conclude that, in real application scenarios, our algorithm improves the efficiency of short-message satellite resource allocation.

## Figures and Tables

**Table 1:** The parameters of feature extraction network.

Layer	Input	Kernel	Activation	Output
Conv1	α×α×26	1×32×26,4	Relu	α×32×4
Conv2	α×32×4	α×16×4,8	Relu	α×16×8
FC1	α×16×8	NA	Relu	1024
FC2	1024	NA	Relu	256
FC3	256	NA	Relu	$14\times \upsilon_{\max}$

**Table 2:** The parameters of the feature extraction network.

Simulation Parameter	Value
Uplink operating frequency/MHz	1620
Downlink operating frequency/MHz	1207.14
Orbital altitude/km	21,528
Total number of short-message terminals	1100~2400
$C_{\max}$/bit	560
τ/s	60
Time taken up by each snapshot/s	60
$n_{t}$	1000
$f_{j}$/Mbit/s	50
$\upsilon_{\max}$	10
Episodes	500
Steps T	500
Batch size	8
Discount factor	0.9
Soft update factor	0.01
Learning rate	0.01
Activation function	Relu
α	64

## Data Availability

Not applicable.

## Abbreviations

**Summary of Main Notations**	
**Symbol**	**Description**
Model	
$SAT=\left\{SAT_{1},SAT_{2},\cdots ,SAT_{n}\right\}$(SAT)	The set of short-message satellites
$ST=\left\{ST_{1},ST_{2},\cdots ,ST_{m}\right\}$(ST)	The set of short-message terminals
n	The number of short-message satellite
m	The number of short-message terminal
t	Snapshoot
$r_{i,j}$	$\mathrm{The}\;\mathrm{communication}\;\mathrm{rate}\;\mathrm{between}\;ST_{i}$$\;\mathrm{and}\;SAT_{j}$.
$A_{n\times n_{t}}$	Resource matrix
$Q^{T}=\left\{q_{1}^{t},q_{2}^{t},\cdots ,q_{\zeta }^{t}\right\}$	The set of task queue
$E_{m\times n}$	The satellite-to-Earth link matrix
L	The path transmission loss
$L_{m\times n}$	The path transmission loss matrix
$L_{total}$	The total path transmission loss
LI	The load balancing index
$SRR_{j}$	$\mathrm{The}\;\mathrm{satellite}\;\mathrm{resource}\;\mathrm{utilization}\;\mathrm{of}\;SAT_{j}$
$Task=\left\{{\mathrm{task}}_{1},task_{2},\cdots ,task_{m}\right\}$	The set of short-message task
SI	The service satisfaction index
U(t)	The objective function
Algorithm	
$A^{r}=\left\{a_{1}^{r},a_{2}^{r},\cdots ,a_{\upsilon }^{r}\right\}$	The set of subregion
$A_{ij}^{d}$	$\mathrm{The}\;\mathrm{regional}\;\mathrm{distance}\;\mathrm{from}\;a_{i}^{r}$ $\;\mathrm{to}\;SAT_{j}$
$d_{\chi j}$	$\mathrm{The}\;\mathrm{distance}\;\mathrm{from}\;ST_{\chi }$ $\;\mathrm{to}\;SAT_{j}$ $\;\mathrm{in}\;a_{i}^{r}$
${\tilde{L}}_{\upsilon \times n}$	$\mathrm{The}\;\mathrm{path}\;\mathrm{transmission}\;\mathrm{loss}\;\mathrm{matrix}\;{\tilde{L}}_{\upsilon \times n}$ between the elements of set SAT $\mathrm{and}\;\mathrm{set}\;A^{r}$
$S=\{s_{1},s_{2},\cdots ,s_{t}\}$	State space
$s_{t}=\left\{L_{t},A_{n\times n_{t}}^{r},LI_{t}\right\}$	The system state under snapshot t
$b_{n}\left(t\right)$	The resource allocation decisions
$a_{t}$	The action under $s_{t}$
$r(s_{t},a_{t})$	The reward function
α	The size of tensor
π	π $\;\mathrm{is}\;\mathrm{transferred}\;\mathrm{from}\;\mathrm{the}\;\mathrm{initial}\;\mathrm{state}\;s_{0}$ $\;\mathrm{to}\;\mathrm{the}\;\mathrm{state}\;s_{t+1}$
$Q_{\pi }^{}\left(s_{t},a_{t}\right)$	The action function
$V_{\pi }\left(s_{t}\right)$	The state function
$Q_{\pi }^{*}\left(s_{t},a_{t}\right)$	The optimal action function
$V^{*}\left(s_{t}\right)$	The optimal state function
$a_{t}^{*}$	The optimal action
J(θ)	The critic network of loss function
J(ω)	The actor network of loss function
ξ	Random noise

## References

[1] Wang M.J., Chen X.X., Wu T., Si D.R., Zhai Z.Z. (2020). Design of integrated radio meteorological parameter monitoring system based on LoRa. Chin. J. Radio Sci..

[2] Wang C.M., Lei W.Y., Huang H., Huang F.L. (2019). Designation of automatic weather station message transmission project based on Beidou. Meteorol. Sci. Technol..

[3] Chen S.T., Lin T., Zhang Y.Y. (2018). Weather warning information transmission method based on Beidou. Chin. J. Electron Devices.

[4] Li H.S., Cao Z.Y., He S.S., Zhou G.Z. (2014). Design and application of meteorological disaster early warning release system based on Beidou Satellite technology. Meteorol. Sci. Technol..

[5] Wang C.F., Chen Y.T., Li C.L., Jiang K.J. (2014). Technology and implementation of warning information distribution based on Beidou satellite. J. Appl. Meteorol. Sci..

[6] Li B.F., Zhang Z.T., Zhang N., Wang S.Y. (2019). High-precision GNSS ocean positioning with BeiDou short-message communication. J. Geod..

[7] Liu D.G., Wu B.G., Xie Y.Y., Luo H.H. (2014). Present state and development trend of maritime meteorological support service. Navig. China.

[8] He K.F., Weng D.J., Ji S.Y., Wang Z.J., Chen W., Lu Y.W. (2020). Ocean Real-Time Precise Point Positioning with the BeiDou Short-Message Service. Remote Sens..

[9] Li G., Guo S.R., Lv J., Zhao K.L., He Z.H. (2021). Introduction to global short message communication service of BeiDou-3 navigation satellite system. Adv. Space Res..

[10] Xiang Y.W., Zhang W.Y., Tian M.M. (2018). Satellite data transmission integrated scheduling and optimization. Syst. Eng. Electron..

[11] Cocco G., Cola T.D., Angelone M., Erl S. (2018). Radio Resource Management Optimization of Flexible Satellite Payloads for DVB-S2 Systems. IEEE Trans. Broadcast..

[12] Zhang P., Wang X.H., Ma Z.G., Song J.D. (2019). Joint optimization of satisfaction index and spectrum efficiency with cache restricted for resource allocation in multi-beam satellite systems. China Commun..

[13] Dai J.H., Liu J.J., Shi Y.P., Zhang S.B., Ma J.F. (2017). Analytical Modeling of Resource Allocation in D2D Overlaying Multihop Multichannel Uplink Cellular Networks. IEEE Trans. Veh. Technol..

[14] Sawyer N., Smith D.B. (2019). Flexible Resource Allocation in Device-to-Device Communications Using Stackelberg Game Theory. IEEE Trans. Commun..

[15] Vakilian V., Frigon J.F., Roy S. (2018). Distributed Resource Allocation for D2D Communications Underlaying Cellular Networks in Time-Varying Environment. IEEE Commun. Lett..

[16] Artiga X., Nunez-Martinez J., Perez-Neira A., Vela G., Garcia J., Ziaragkas G. Terrestrial-satellite integration in dynamic 5G backhaul networks. Proceedings of the 2016 8th Advanced Satellite Multimedia Systems Conference and the 14th Signal Processing for Space Communications Workshop (ASMS/SPSC).

[17] Choi J.P., Chan V.W.S. (2005). Optimum power and beam allocation based on traffic demands and channel conditions over satellite downlinks. IEEE Trans. Wirel. Commun..

[18] Kan X., Xu X.D. (2016). Power allocation based on energy and spectral efficiency in multi-beam satellite systems. J. Univ. Sci. Technol. China.

[19] Aravanis A.I., Shankar M.R.B., Arapoglou P.D., Danoy G., Cottis P.G., Ottersten B. (2015). Power Allocation in Multibeam Satellite Systems: A Two-Stage Multi-Objective Optimization. IEEE Trans. Wirel. Commun..

[20] Efrem C.N., Panagopoulos A.D. (2020). Dynamic Energy-Efficient Power Allocation in Multibeam Satellite Systems. IEEE Wirel. Commun. Lett..

[21] Jiao J., Sun Y.Y., Wu S.H. (2020). Network Utility Maximization Resource Allocation for NOMA in Satellite-Based Internet of Things. IEEE Internet Things J..

[22] Lin C.C., Su N.W., Deng D.J., Tsai I.H. (2020). Resource allocation of simultaneous wireless information and power transmission of multi-beam solar power satellites in space–terrestrial integrated networks for 6G wireless systems. Wirel. Netw..

[23] Yang L., Yang H., Wei D.B., Pan C.S. (2020). SAGA: A Task-oriented Resource Allocation Algorithms for Satellite Network. J. Chin. Comput. Syst..

[24] Xia K.W., Feng J., Wang Q.R., Yan C. Optimal Selection Mechanism of Short Message Terminal for “Beidou-3”. Proceedings of the 2020 IEEE 5th International Conference on Signal and Image Processing (ICSIP).

[25] Liu Q., Zhai J.W., Zhang Z.C., Zhong S., Zhou Q., Zhang P., Xu J. (2018). A survey on deep reinforcement learning. Chin. J. Comput..

[26] Gu B., Zhang X., Lin Z., Alazab M. (2021). Deep Multiagent Reinforcement-Learning-Based Resource Allocation for Internet of Controllable Things. IEEE Internet Things J..

[27] Zhao N., Liang Y., Niyato D., Pei Y., Wu M., Jiang Y. (2019). Deep Reinforcement Learning for User Association and Resource Allocation in Heterogeneous Cellular Networks. IEEE Trans. Wirel. Commun..

[28] Tang F., Zhou Y., Kato N. (2020). Deep Reinforcement Learning for Dynamic Uplink/Downlink Resource Allocation in High Mobility 5G HetNet. IEEE J. Sel. Areas Commun..

[29] Hu X., Zhang Y., Liao X., Liu Z., Wang W., Ghannouchi F.M. (2020). Dynamic Beam Hopping Method Based on Multi-Objective Deep Reinforcement Learning for Next Generation Satellite Broadband Systems. IEEE Trans. Broadcast..

[30] Xiong X., Zheng K., Lei L., Hou L. (2020). Resource Allocation Based on Deep Reinforcement Learning in IoT Edge Computing. IEEE J. Sel. Areas Commun..

[31] Takahashi M., Kawamoto Y., Kato N., Miura A., Toyoshima M. (2019). Adaptive Power Resource Allocation with Multi-Beam Directivity Control in High-Throughput Satellite Communication System. IEEE Wirel. Commun. Lett..

[32] Ferreira P., Paffenroth R., Wyglinski A.M. (2018). Multiobjective Reinforcement Learning for Cognitive Satellite Communications Using Deep Neural Network Ensembles. IEEE J. Sel. Areas Commun..

[33] Hu X., Liu S., Chen R., Wang W., Wang C. (2018). A Deep Reinforcement Learning-Based Framework for Dynamic Resource Allocation in Multibeam Satellite Systems. IEEE Commun. Lett..

[34] Hu X., Liu S., Wang Y., Xu L., Zhang Y., Wang C., Wang W. (2019). Deep reinforcement learning based beam hopping algorithm in multibeam satellite systems. IET Commun..

[35] Luis J.J.G., Guerster M., del Portillo I., Crawley E., Cameron B. Deep Reinforcement Learning for Continuous Power Allocation in Flexible High Throughput Satellites. Proceedings of the 2019 IEEE Cognitive Communications for Aerospace Applications Workshop (CCAAW).

[36] Yu Z., Machado P., Zahid A., Abdulghani A.M., Dashtipour K., Heidari H., Imran M.A., Abbasi Q.H. (2020). Energy and performance trade-off optimization in heterogeneous computing via reinforcement learning. Electronics.

[37] Zhang P., Liu S.J., Ma Z.G., Wang X.H., Song D.J. (2020). Improved satellite resource allocation algorithmbased on DRL and MOP. J. Commun..

[38] Qiu C., Yao H., Yu F.R., Xu F., Zhao C. (2019). Deep Q-Learning Aided Networking, Caching, and Computing Resources Allocation in Software-Defined Satellite-Terrestrial Networks. IEEE Trans. Veh. Technol..

[39] China Satellite Navigation Office BeiDou Navigation Satellite System Open Service Performance Standard [EB/OL]. 2018-12-28. http://www.beidou.gov.cn.

[40] Gounder V.V., Prakash R., Abu-Amara H. Routing in LEO-based satellite networks. Proceedings of the 1999 IEEE Emerging Technologies Symposium, Wireless Communications and Systems (IEEE Cat. No.99EX297).

[41] Ministry of Industry and Information Technology of the People’s Republic of China (2020). Methods for Calculating Attanuations by Atmospheric Gases and Rain in the Satellite Communication Link (YD/T 984-2020).

[42] Chen L.M., Guo Q., Yang M.C. (2012). Probability-Based Bandwidth Reservation Strategy for LEO Satellite Networks with Multi-Class Traffic. J. South China Univ. Technol. (Nat. Sci. Ed.).

[43] Yang B., He F., Jin J., Xu G.H. (2014). Analysis of Coverage Time and Handoff Number on LEO Satellite Communication Systems. J. Electron. Inf. Technol..

[44] Xu S.Y., Xing Y.F., Guo S.G., Yang C., Qiu X.S., Meng L.M. (2021). Deep reinforcement learning based task allocation mechanism for intelligent inspection in energy Internet. J. Commun..

